# Assessing the three attentional networks in children from three to six years: A child-friendly version of the Attentional Network Test for Interaction

**DOI:** 10.3758/s13428-021-01668-5

**Published:** 2021-09-30

**Authors:** Maria Casagrande, Andrea Marotta, Diana Martella, Elisa Volpari, Francesca Agostini, Francesca Favieri, Giuseppe Forte, Monica Rea, Rosa Ferri, Vito Giordano, Fabrizio Doricchi, Jasmine Giovannoli

**Affiliations:** 1 Dipartimento di Psicologia Dinamica, Clinica e Salute, Università di Roma “Sapienza”, Rome, Italy; 2 Departamento de Psicología Experimental, Universidad de Granada, Granada, Spain; 3 Instituto de Estudios Sociales y Humanísticos, Universidad Autónoma de Chile, Santiago de Chile, Chile; 4 Istituto di Istruzione Superiore V. Bachelet, Abbiategrasso, Italy; 5 Dipartimento di Psicologia, Università di Roma “Sapienza”, Roma, Italy; 6 Department of Pediatrics and Adolescent Medicine, Division of Neonatology, Pediatric Intensive Care and Neuropediatrics, Comprehensive Center for Pediatrics, Medical University of Vienna, Vienna, Austria

**Keywords:** Attention, Alerting, Orienting, Executive Control, ANTI, Children

## Abstract

Attention involves three functionally and neuroanatomically distinct neural networks: alerting, orienting, and executive control. This study aimed to analyze the development of attentional networks in children aged between 3 and 6 years using a child-friendly version of the Attentional Network Test for Interaction (ANTI), the ANTI-Birds. The sample included 88 children divided into four age groups: 3-year-old, 4-year-old, 5-year-old, 6-year-old children. The results of this study would seem to indicate that between 4 and 6 years, there are no significant changes in attentional networks. Instead, between 3 and 4 years of age, children significantly improve all their attentional skills.

## Introduction

Attention processes are essential to efficient functioning in everyday life and for the individual's academic and general success. Attentional diseases are common in a range of genetic, developmental, and acquired disorders in children. Given the impact of such problems, understanding attention processes during early development is essential (Atkinson & Braddick, 2012).

Attention during childhood has been extensively investigated (e.g., Iarocci et al., 2009; Marotta & Casagrande, 2016; Ridderinkhof & van der Stelt, 2000; Scerif, 2010;) and developmental changes, and child–adult differences have been generally reported. To understand what these developmental differences might mean, it is useful to consider the neurocognitive model of attention proposed by Posner and Petersen (Petersen & Posner, 2012; Posner & Petersen, 1990). According to this model, attention involves three neural networks: alerting, orienting, and executive control. The alerting network aims to achieve (phasic alerting) and maintain (tonic alerting or vigilance) a general state of activation of the cognitive systems. The orienting network supports the ability to select and focus on specific information. The executive network manages the ability to solve incongruent information.

To assess the efficiency of the three attentional networks, Fan et al. (2002) developed the Attentional Networks Test (ANT), combining the Cued Reaction Time (Posner, 1980) and the flanker paradigm (Eriksen & Eriksen, 1974). The ANT allows evaluating simultaneously and rapidly the three attentional networks. These aspects allowed the use of the ANT in different research contexts and different populations such as adults (e.g., Federico et al., 2013; Martella et al., 2011; Spagna et al., 2014, 2016), older adults (e.g., Casagrande et al., 2021; Federico et al., 2021), adolescents (e.g., Casagrande et al., 2017; Giovannoli et al., 2021), children (e.g., Federico et al., 2017; Mezzacappa, 2004; Rueda et al., 2004) and also clinical populations (e.g., Casagrande et al., 2012; Marotta et al., 2015).

In the classic version of the ANT, alerting and orienting are measured with the same variable (visual cue); moreover, the spatial cue is 100% predictive (i.e., the cue appears in the same location as the target), and it does not allow evaluating the reorienting of attention due to invalid cues. The task design does not allow assessing the exogenous (i.e., involuntary, bottom-up, stimulus-driven) and endogenous (i.e., voluntary, top-down, goal-driven) components of attention independently. To overcome these limits, Callejas et al. (2005) designed a new version of this task, the Attentional Network Test for Interaction (ANTI). In the ANTI, the double cue was replaced with an alerting tone (auditory warning) presented in 50% of the trials; further, the percentage of predictivity of the spatial cue was manipulated, including one-third of valid trials, one-third of no cue trials, and one-third of invalid trials. This new structure of the task allows independently assessing the three networks and their interactions.

Another criticism was relative to the nature of the stimuli. Accordingly, some studies explored whether the characteristics of the stimuli can modulate the performance in the ANT or ANTI (Boncompagni & Casagrande, 2019; Federico et al., 2013, 2017, 2020; Roca et al., 2011; Rueda et al., 2004; Spagna et al., 2014, 2016). Specifically, Spagna et al. (2014) investigated the attentional effects in response to non-directional and directional stimuli. The results suggested that directional stimuli make the task more difficult, increasing the interaction between conflict and orienting networks. Moreover, target-flanker similarity could also contribute to an interference or facilitation effect (e.g., Moore et al., 2021; Sanders & Lamers, 2002).

Although children can complete the classic version of the ANT, Rueda et al. (2004) designed a child-friendly version of this task. In this version, one or five fishes replaced the classic target (arrow) of the ANT to make it more appealing to children. Furthermore, the experimental task was introduced by a story in which participants had to feed a hungry fish. Auditory and visual feedback informed children about their performances. These changes were made because children's performance and motivation improve when there is a story, and they receive clear feedback (Casagrande et al., 2012; Luman et al., 2005).

The developmental period ranging from 6 to 13 years seems critical for behavioral and brain development (Casey et al., 2005; Rueda et al., 2004). Several cognitive functions (e.g., response inhibition, attentional disengagement, error monitoring) develop between 6 and 9 years of age (Gupta & Kar, 2009).

Focusing on attention, Rueda et al. (2004) showed that reaction times and accuracy improve between 6 and 9 years old. However, different results were reported considering individual attentional networks. Alerting is present in infancy (Colombo, 2001) but continues to develop during childhood (Mezzacappa, 2004; Rueda et al., 2004). Comparing children’s speed of responding to targets with and without visual warning cues a trend to larger alerting scores with age in a sample of 5 to 7-year-olds (Mezzacappa, 2004) and 10-year-olds displayed larger alerting effects than adults (Rueda et al., 2004). On the other hand, an attention-training study by Rueda et al. (2012), including 5-year-old children, failed to find robust alerting effects in the pre-test since both control and training groups displayed very small effects for the alerting network. Conversely, later studies (Abundis-Gutiérrez et al., 2014; Pozuelos et al., 2014) observed larger alerting effects (i.e., larger benefit from having a warning tone before the presentation of the target) in children between 4 and 6 years, suggesting that this result could be related to greater difficulty in maintaining an optimal state of vigilance in the absence of warning cues. Moreover, ERP analysis revealed poor early processing of warning tone in early and middle childhood (Abundis-Gutiérrez et al., 2014).

The orienting network is believed to be stable during infancy and adulthood (from age 6; Gupta & Kar, 2009; Ishigami & Klein, 2015; Rueda et al., 2004), but it has a peak during adolescence (Mezzacappa, 2004). However, research on preschoolers has suggested that the results of the cueing conditions may not be as straightforward as initially claimed (Rueda et al., 2004). Indeed, in the above-mentioned attention-training study by Rueda et al. (2012), several observed orienting scores were negative or just slightly positive. The not-significant orienting effect obtained in the ANT seems to indicate that preschool children may not interpret and utilize the information provided by the spatial cue in this type of task. Moreover, no clear indications were reported about reorienting attention (i.e., reallocating attentional resources to relevant stimuli). Some studies hypothesize that this network develops later than the orienting network (e.g., Abundis-Gutiérrez et al., 2014), while others suggest that it follows the same evolutive pattern (Lewis et al., 2018).

Executive control and the ability to solve incongruent information improve drastically between 6 and 7 years (Rothbart, 2007; Rueda et al., 2004) and reach a level similar to that of adults between the ages of 8 and 10 (Mezzacappa, 2004). Similar results have been observed in several studies, in which consistent large cognitive control scores have been shown in young children (Forns et al., 2014; Pizzo et al., 2010; Rueda et al., 2012).

Although the ANT is generally recommended for use with children as young as 4 years of age, only a few studies have focused on this particular age group and, to our knowledge, no study has used the ANT to evaluate children under the age of 4. Moreover, some studies that have included a preschool sample have found inconsistent results (Forns et al., 2014; Ishigami & Klein, 2015; Rueda et al., 2012).

For this purpose, a useful task is the ANT-child (Rueda et al., 2004), but this test has the same limitations as the original version of the ANT (i.e., it does not independently evaluate the alerting and orienting systems and includes valid trials only). To overcome these limits, some study used a child version of ANTI (Abundis-Gutiérrez et al., 2014; Pozuelos et al., 2014). However, directional stimuli can imply an excessive involvement of the executive system, making the task too difficult for children (e.g., Spagna et al., 2014). Furthermore, to assess the attentional networks of children from 8 years of age could be used AttentionTrip, a video game-like task based on the original ANT (Arora et al., 2021; Klein et al., 2017). In the present study, we used a child-friendly variant of the ANTI task, the ANTI-Birds, similar in its characteristics (colored stimuli, storytelling, feedback) to the ANT-child, but using non-directional stimuli. In particular, the classic target was replaced by a non-directional stimulus (i.e., a bird), and the request was to detect the color of the stimulus. Furthermore, participants received positive or negative feedback for each trial to improve their motivation and performances. Similarly to ANT-child, the task was presented as a story.

To the best of our knowledge, this is the first study to examine the development and interactions among attention networks in children aged 3 through 6 years using the ANTI task with non-directional stimuli. Therefore, this study will be useful to examine developmental effects eventually present in early childhood and provide new information about how attention networks interact. Since the ANTI-Birds had not previously been used, our first aim was to evaluate whether this task can measure the three attention networks in this age range. We expected a performance improvement (in terms of accuracy and reaction times) with advancing age. Given that previous studies have found 4- and 5-year-olds children to have difficulties improving performance using spatial and auditory cues, we expect a lower ability to use visual and warning cues to enhance their performance and progressive improvement in these skills in the older age groups. We also expect a higher adverse effect of incongruent information by younger children and progressive improvement with advancing age.

## Method

### Participants

Eighty-eight children participated in the study. The participants were divided into four age groups: 3-year-old (mean age 45.67 ± 1.53 months), 4-year-old (mean age 54.40 ± 2.07 months), 5-year-old (mean age 69.33 ± 0.58 months), 6-year-old children (mean age 73.00 ± 1.41 months). Each group consisted of 22 participants (eight girls, 14 boys). Children were recruited in Italian public schools. All participants had normal or correct-to-normal vision, and no one was color blind. Children with prior history of mental retardation, brain trauma, neurological disease, physical impairment, and learning disabilities were excluded. The ethics committee of the Department of Dynamic and Clinical Psychology at the University of Rome "Sapienza" approved the project. The parents of the participants signed informed consent for the participation of the children in the research.

### Apparatus

The experiment was programmed and displayed by E-Prime software (Schneider et al., 2002) on an Intel Core i5 PC and displayed on a 15-inch color screen. Participants viewed the screen from about 56 cm. The participant’s responses were recorded through a standard mouse, and the acoustic tone was presented through headphones.

### ANTI-Birds

#### Stimuli

Each trial began with the presentation of a central nest measuring 3° (degrees of visual angle), used as a fixation point over a background representing a tree. A horizontal row of five yellow eggs was presented above and below fixation. A single egg consisted of 1° of the visual field, and the contours of adjacent eggs were separated by 0.2° of the visual field. The target was a yellow or orange bird included in a horizontal row of five birds. The stimuli (one central bird plus four flankers) subtended a total of 6.5° of the visual field. The target and flankers were presented above or below the fixation point. The task was to identify the color of the centrally presented bird by clicking the right (yellow bird) or left (orange bird) button on the mouse. On congruent trials, the target was an orange or yellow bird flanked on both sides by two birds of the same color. On incongruent trials, the flanker birds were of a different color. The target and flankers were presented at 1.5° above or below the fixation point. The cue consisted of a thick contour of the eggs, and it could be presented at the position of the upcoming target (valid condition), in the opposite location (invalid condition), or it could be absent (no cue condition). The auditory warning stimulus was 2000 Hz and lasted 200 ms. For correct responses, the visual feedback was a drawing showing several flying birds with the message “Correct!” and auditory feedback of birds chirping. For incorrect responses, the visual feedback was a nest with two sad birds over grey background with the message “Incorrect!” and auditory feedback of people saying “Noooo!”.

#### Procedure

Children were tested individually in a silent and dimly illuminated room. Each trial began with a fixation period of variable duration (400–1600 ms), and this was followed by a warning stimulus lasting 200 ms in 50% of the trials. After a fixation period of 350 ms, a cue of 100 ms was presented. In the valid condition (33% of the trials), the visual cue appeared in the same location as the target; in the invalid condition (33%), it was presented in the opposite location; in the no-cue condition (33%), no orienting stimulus was presented. After a variable interstimulus interval (ISI, 100–150 ms), the target was presented until the participant responded, with a limit of 2000 ms. Two birds flanked the central bird on each side that could be of the same color (congruent condition, 50% of the trials) or a different color (incongruent condition). After responding, the participants received visual and auditory feedback for correct and incorrect responses. Then, the trial ends with a fixation period of 250 ms. The fixation point was at the center of the screen throughout the trials. Children were instructed to fixate the central nest and respond to the target as quickly and accurately as possible.

The task consisted of a 12-trial practice block and three experimental blocks of 48 trials. The entire experiment included 144 experimental trials. The trials were presented randomly within each block.

**Fig. 1:**
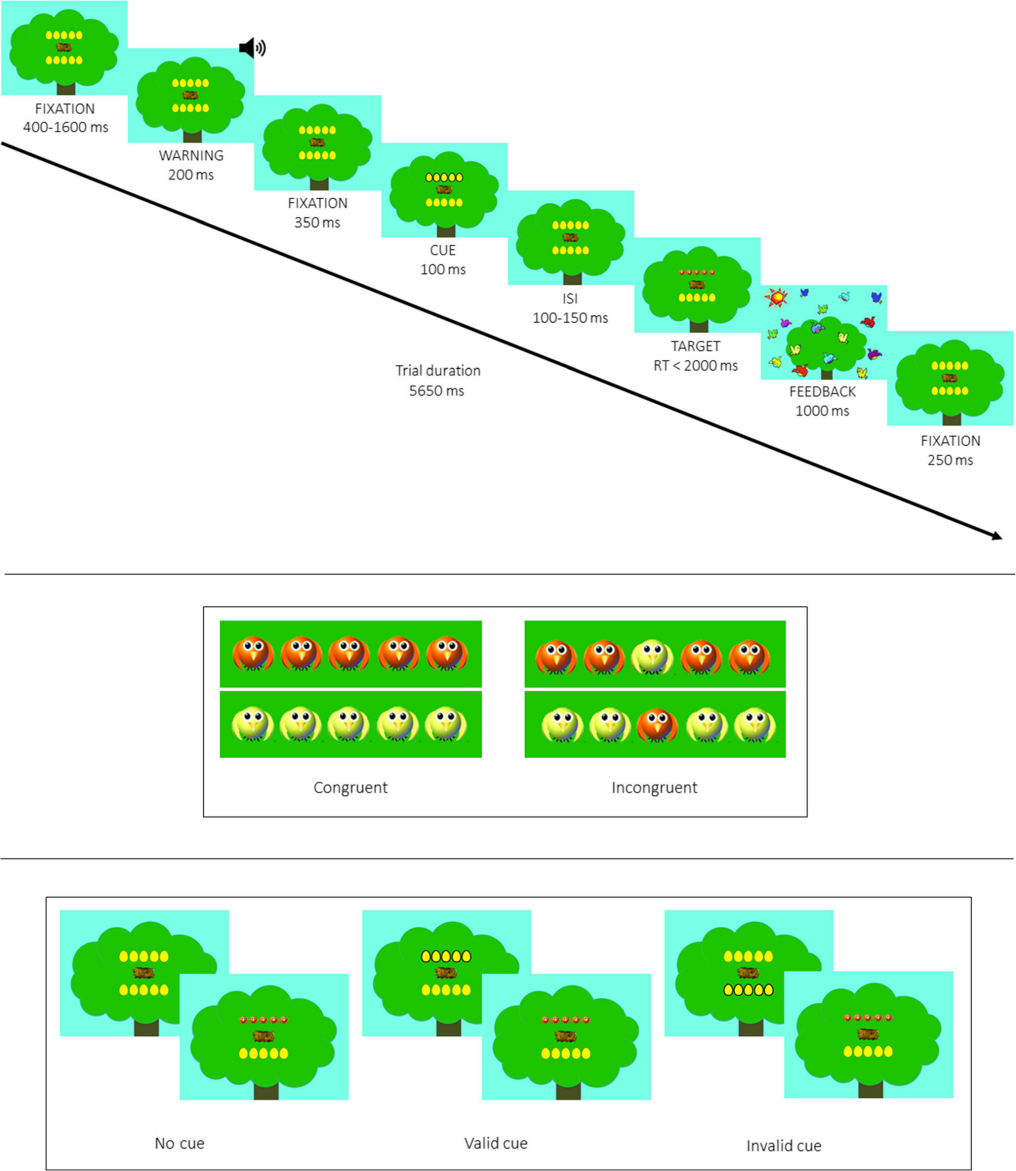
Schematic representation of the procedure, target stimuli and experimental conditions

The children could take breaks at the end of the practice block and between test blocks. The practice block lasted around 1 min, and each test block took approximately 6 min. The task lasted about 20 min. The sequence of the experimental procedure for each trial is shown in Fig. 1.

### Statistical Analysis

A Group (3-years-old, 4-years-old. 5-years-old, 6-years-old) x Warning (Warning, No-Warning) x Cue (Valid, Invalid, No-cue) x Executive control (Congruent, Incongruent) mixed analysis of variance (ANOVA) was conducted on both the median RTs of the correct responses and the mean percentage of accuracy.

Furthermore, some attentional effects were computed as a subtraction from specific conditions: a) Alerting effect: no-warning - warning conditions; b) Orienting effect: invalid - valid conditions; c) Executive control: incongruent - congruent conditions; d) Attentional costs: invalid trials – no-cue trials, and e) Attentional benefits: no-cue trials - valid trials.

The alerting effect represents the benefit of a warning stimulus on the speed of the response to the target (Callejas et al., 2005).

Visual cues provide a measure of orienting attention. Both invalid and valid cues alert the participant to the forthcoming appearance of the target, but only the valid cue provides spatial information, which allows participants to orient their attention to the appropriate spatial location.

The difference between median RTs in incongruent and congruent trials was considered an index of executive control because the two conditions differ only in the information given by the flankers. When the images are congruent, they facilitate the discrimination of the target stimulus, whereas incongruent flankers distract participants (Fan et al., 2002).

Higher costs reflect the price due to disengaging attention from an invalid position, while higher benefits represent the orienting advantage of having a valid spatial cue.

One-way ANOVAs on Alerting, Orienting, Executive Control, Attentional costs, and Attentional benefits effects were conducted to estimate the efficiency of each attentional system.

To assess the effect of target color a Group (3-years-old, 4-years-old. 5-years-old, 6-years-old) x Target Color (Orange, Yellow) x Warning (Warning, No-Warning) x Cue (Valid, Invalid, No-cue) x Executive control (Congruent, Incongruent) mixed analysis of variance (ANOVA) was conducted on both the median RTs of the correct responses and the mean percentage of accuracy.

Planned comparisons were used to analyze the main effects of the task and the interactions.

For each network component, the split-half reliability analysis was conducted using the plyr (Wickham, 2011) and Hmisc (Harrell, 2018) libraries in R Studio (R Core Team, 2018). According to Roca et al. (2018) and Luna et al. (2021), all the experimental trials of each participant were randomly split into two halves considering RT and the attentional effects (Alerting, Orienting, Executive Control). The permutation approach (i.e., 10,000 pairs of trial halves) was adopted. The split-half reliability indices were obtained as the average of the 10,000 Pearson’s r correlations for each attentional network and general RTs. Finally, the correlation was adjusted and attenuated for length by the Spearman–Brown formula (Spearman, 1910) for the split-half reliability (MacLeod et al., 2010; Luna et al., 2021). The size of reliability indices was interpreted following Draheim et al. (2019): below .70 as problematic, between .70 and .79 as borderline, and above .80 as acceptable.

An α value of 0.05 was used to establish statistical significance for all analyses. Data were analyzed using Statistica (StatSoft, Inc Tulsa, OK) v. 10.

## Power analysis

A power analysis using G*Power 3.1 (Faul et al., 2007) was conducted. We assumed medium-sized effects to assess interactions between groups and each attention network. The analysis indicated that the current sample size was sufficient to detect these effects at a power of > 80% with a type 1 error (α < .05).

## Results

Table 1 shows the complete ANOVA results. Table 2 shows the median reaction times for correct responses and the accuracy percentage for each condition and age group. All analyses were initially conducted with gender as a between-group factor, but the main effect of gender and its interactions were not significant; therefore, the gender was collapsed.

**Table 1:** ANOVA results

	*df*	F	*p*	η^2^
Reaction times				
Group	3,84	12.29	< .01	.30
Warning	1,84	35.90	< .01	.30
Warning x Group	3,84	3.05	.03	.10
Executive Control	1,84	130.24	< .01	.61
Executive Control x Group	3,84	5.18	< .01	.16
Cue	2,168	59.79	< .01	.42
Cue x Group	6,168	4.95	< .01	.15
Warning x Executive Control	1,84	0.60	.44	.01
Warning x Executive Control x Group	3,84	.28	.84	.01
Warning x Cue	1,84	.60	.55	.01
Warning x Cue x Group	3,84	.85	.54	.03
Executive Control x Cue	2,168	.83	.44	.01
Executive Control x Cue x Group	6,168	1.47	.19	.05
Warning x Executive Control x Cue	2,168	.37	.69	.004
Warning x Executive Control x Cue x Group	6,168	.75	.61	.03
Alerting	3,84	3.05	.03	.10
Orienting	3,84	4.18	< .01	.13
Executive Control	3,84	5.18	< .01	.16
Costs	3,84	8.04	< .01	.22
Benefits	3,84	2.15	.10	.07
Accuracy				
Group	3,84	13.98	< .01	.33
Warning	1,84	7.37	< .01	.08
Warning x Group	3,84	0.15	.93	.005
Executive Control	1,84	23.72	< .01	.22
Executive Control x Group	3,84	0.77	.51	.03
Cue	2,168	1.28	.28	.01
Cue x Group	6,168	2.22	.04	.07
Warning x Executive Control	1,84	0.83	.36	.01
Warning x Executive Control x Group	3,84	0.73	.54	.02
Warning x Cue	2,168	0.03	.97	< .01
Warning x Cue x Group	6,168	0.48	.82	.02
Executive Control x Cue	2,168	0.24	.79	.003
Executive Control x Cue x Group	6,168	0.53	.79	.02
Warning x Executive Control x Cue	2,168	0.23	.80	.003
Warning x Executive Control x Cue x Group	6,168	1.35	.24	.05
Alerting	3,84	.15	.93	.005
Orienting	3,84	3.69	.01	.12
Executive Control	3,84	.77	.51	.03
Costs	3,84	1.04	.38	.04
Benefits	3,84	1.76	.16	.06

**Table 2:** Median reaction times (in milliseconds) and percentage of accuracy for each age group and in all experimental conditions

			3-year-old (*n* =22)		4-year-old (*n* = 22)		5-year-old (*n* = 22)		6-year-old (*n* = 22)	
			RTs	Accuracy	RTs	Accuracy	RTs	Accuracy	RTs	Accuracy
No warning	Congruent	Valid	764.16 (217.95)	78.82 (13.28)	1110.25 (225.70)	81.86 (13.09)	980.57 (147.27)	96.68 (4.85)	962.14 (151.66)	92.45 (6.88)
		Invalid	805.34 (248.86)	86.82 (10.82)	1182.16 (267.48)	86.09 (14.60)	1046.82 (142.96)	93.64 (7.28)	1052.59 (170.77)	90.95 (7.35)
		No Cue	911.66 (259.78)	80.68 (13.47)	1164.61 (197.48)	86.36 (11.72)	1055.82 (196.37)	94.00 (7.39)	1010.25 (154.72)	89.50 (10.36)
	Incongruent	Valid	836.50 (369.17)	77.27 (17.80)	1198.11 (255.51)	78.36 (16.31)	1122.73 (136.21)	92.04 (7.17)	1088.86 (224.94)	87.54 (16.20)
		Invalid	826.25 (336.24)	78.73 (19.42)	1324.02 (267.50)	78.41 (18.72)	1218.66 (140.51)	91.36 (8.36)	1162.61 (243.00)	87.59 (11.51)
		No Cue	917.66 (310.85)	78.86 (16.77)	1310.50 (252.93)	78.09 (12.23)	1190.91 (149.59)	87.86 (10.84)	1131.50 (167.99)	89.09 (15.52)
Warning	Congruent	Valid	735.14 (196.23)	80.64 (11.57)	1037.02 (216.47)	86.45 (11.91)	921.98 (134.05)	93.68 (6.78)	930.82 (162.23)	93.27 (9.11)
		Invalid	802.16 (252.38)	84.91 (10.20)	1148.43 (211.75)	86.00 (9.45)	1011.41 (134.58)	93.64 (8.49)	987.20 (157.63)	94.41 (6.99)
		No Cue	879.27 (268.84)	83.04 (13.00)	1100.82 (194.93)	85.18 (10.97)	1001.18 (155.86)	94.77 (6.09)	982.93 (180.25)	92.86 (9.37)
	Incongruent	Valid	849.41 (355.11)	76.45 (17.58)	1192.66 (211.90)	80.73 (15.68)	1047.41 (126.57)	94.41 (6.99)	1056.04 (190.45)	90.18 (9.87)
		Invalid	873.36 (349.71)	85.64 (12.16)	1244.45 (208.49)	79.64 (9.83)	1188.95 (114.12)	92.83 (8.70)	1128.54 (245.07)	88.68 (10.27)
		No Cue	878.52 (299.13)	80.64 (17.57)	1224.68 (219.19)	82.64 (11.72)	1149.11 (128.75)	91.73 (8.20)	1104.11 (175.08)	87.09 (10.93)

*Note.* Standard deviations are in parentheses. RTs: reaction times

### Reaction time analysis

**Fig. 2:**
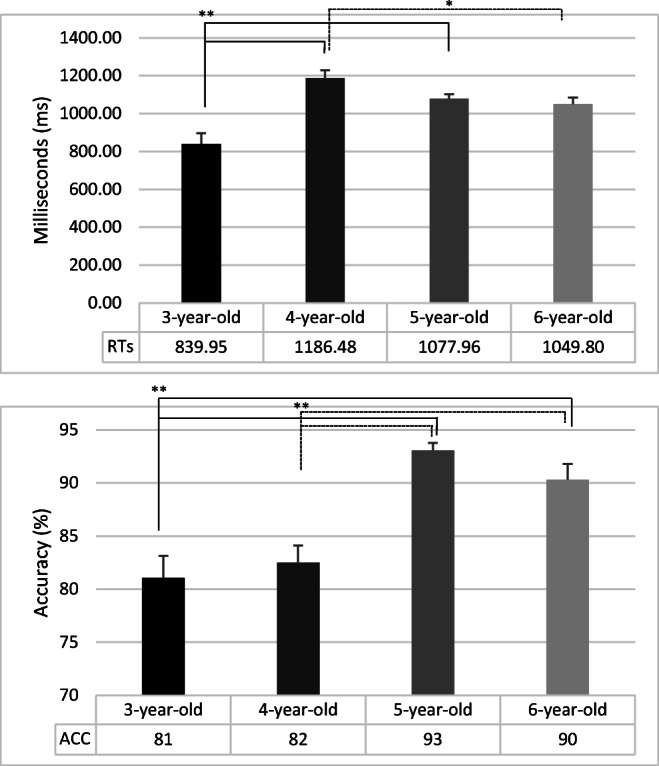
Mean reaction times and accuracy of for each group of children. *Error bars* represent standard errors. *p < .05; **p < .01

The analysis of variance showed significant main effects of Group (F_3,84_ = 12.29; *p* < .01; η^2^ = .30), Warning (F_1,84_ = 35.89; *p* < .01; η^2^ = .30), Cue (F_2,168_ = 59.79; *p* < .01; η^2^ = .42), and Executive control (F_1,84_ = 130.24; *p* < .01; η^2^ = .61). Children of 3 years old were faster than children of 4- (F_1,84_ = 35.13; *p* < .01; η^2^ = .29), 5 years (F_1,84_ = 16.57; *p* < .01; η^2^ = .16) and 6-year-old (F_1,84_ = 12.88; *p* < .01; η^2^ = .13). Children of 4 years old did not differ from children of 5 years old (F_1,84_ = 3.44; *p* = .07; η^2^ = .04), while were slower than children of 6 years old (F_1,84_ = 5.46; *p* = .02; η^2^ = .06). No other between differences were significant (see Fig. 2).

Participants were faster when an auditory signal was provided and in the congruent trials. Furthermore, participants were faster in the valid trials respect to invalid (F_1,84_ = 97.52; *p <* .01; η^2^ = .54) and no-cue trials (F_1,84_ = 92.79; *p <* .01; η^2^ = .52) while no significant difference emerged in no-cue respect to invalid trials (F < 1).

Considering the interactions, Group x Cue (F_6,168_ = 4.95; *p <* .01; η^2^ = .15), Group x Executive control (F_3,84_ = 5.18; *p <* .01; η^2^ = .16) and Group x Warning (F_3,84_ = 3.05; *p* = .03; η^2^ = .10) were significant. Specifically, considering Warning condition children of 3 years old were faster than all other age groups in both warning (4 years: F_1,84_ = 31.29; *p <* .01; η^2^ = .27; 5 years: F_1,84_ = 14.24; *p <* .01; η^2^ = .14; 6 years: F_1,84_ = 11.53; *p <* .01; η^2^ = .12) and no-warning conditions (4 years: F_1,84_ = 37.41 *p <* .01; η^2^ = .31; 5 years: F_1,84_ = 18.20; *p <* .01; η^2^ = .18; 6 years: F_1,84_ = 13.66; *p <* .01; η^2^ = .14). Children of 4 years old were slower than children of 6 years old in both conditions (no warning: F_1,84_ = 8.86; *p* = .02; η^2^ = .07; warning: F_1,84_ = 4.83; *p* = .03; η^2^ = .05). There were no other significant differences (*p* > .05). Moreover, 4-, 5- and 6-year-old children responded faster in warning trials respect to no-warning trials (4 years: F_1,84_ = 20.73; *p <* .01; η^2^ = .20; 5 years: F_1,84_ = 15.51; *p <* .01; η^2^ = .16; 6 years: F_1,84_ = 8.46; *p <* .01; η^2^ = .09), while no difference was found in 3 years children (F < 1).

Regarding Cue, all groups were faster in valid trials than invalid trials (3 years: F_1,84_ = 4.24; *p* = .04; η^2^ = .05; 4 years: F_1,84_ = 37.19; *p <* .01; η^2^ = .31; 5 years: F_1,84_ = 44.11; *p <* .01; η^2^ = .34; 6 years: F_1,84_ = 24.51; *p <* .01; η^2^ = .23). Additionally, children of 3 years old were faster than all other age groups in invalid trials (4 years: F_1,84_ = 38.94; *p* < .01; η^2^ = .32; 5 years: F_1,84_ = 20.63; *p* < .01; η^2^ = .20; 6 years: F_1,84_ = 16.10; *p* < .01; η^2^ = .16), valid trials (4 years F_1,84_ = 33.79; *p* < .01; η^2^ = .29; 5 years: F_1,84_ = 14.54; *p <* .01; η^2^ = .15; 6 years: F_1,84_ = 13.42; *p <* .01; η^2^ = .14) and no-cue trials (4 years: F_1,84_ = 28.04; *p <* .01; η^2^ = .25; 5 years: F_1,84_ = 12.49; *p <* .01; η^2^ = .13; 6 years: F_1,84_ = 7.84; *p <* .01; η^2^ = .09). In valid trials, children of 4 years old were slower than children of 5 years old (F_1,84_ = 4.00; *p* = .05; η^2^ = .05). In all cue conditions, children of 4 years old were slower than children of 6 years old (invalid cue: F_1,84_ = 4.96; *p* = .03; η^2^ = .06; no cue F_1,84_ = 6.23; *p* = .01; η^2^ = .07; valid cue: F_1,84_ = 4.62; *p* = .03; η^2^ = .05).

**Fig. 3:**
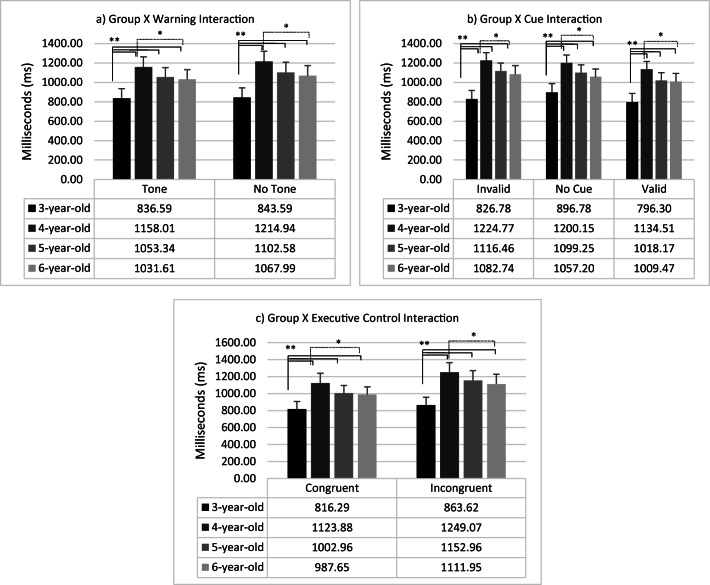
Intera*ctions between Group and* **a** *Warning,* **b** *Cue, and* **c** Executive control. *Error bars* represent standard errors. *p < .05; **p < .01

Considering Executive control interaction, children of 3 years old were faster than other groups in congruent trials (4 years: F_1,84_ = 34.12; *p <* .01; η^2^ = .29; 5 years: F_1,84_ = 12.56; *p <* .01; η^2^ = .13; 6 years: F_1,84_ = 10.59; *p <* .01; η^2^ = .11). Moreover, children of 4 years old were slower than children of 5 years old (F_1,84_ = 5.27; *p* = .02; η^2^ = .06) and 6 years (F_1,84_ = 6.69; *p* = .01; η^2^ = .07). No other differences were found (F < 1). In incongruent trial, children of 3 years old were faster than other groups (4 years: F_1,84_ = 33.41; *p <* .01; η^2^ = .28; 5 years: F_1,84_ = 18.83; *p <* .01; η^2^ = .18; 6 years: F_1,84_ = 13.87; *p <* .01; η^2^ = .14). Children of 4 years old were slower than children of 6 years old (F_1,84_ = 4.23; *p* = .04; η^2^ = .05). Figure 3 reported the interaction of the Group with the Warning, the Cue and the Executive control conditions.

### Attentional effects

**Fig. 4:**
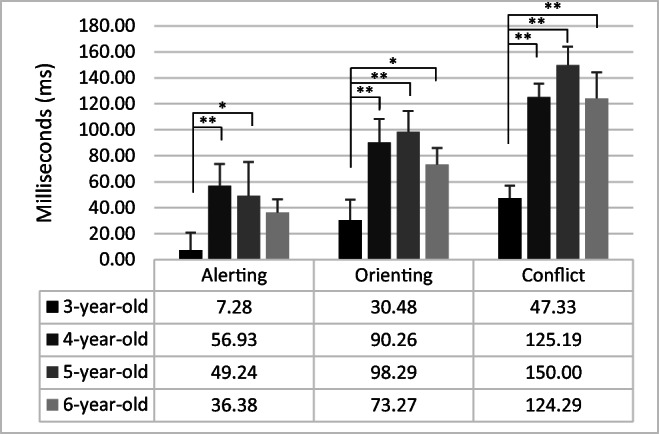
Attentional effects for each group of children. *Error bars* represent standard errors. *p < .05; **p < .01

One-way ANOVAs revealed a significant effect of Group for Alerting (F_3,84_ = 3.05; *p* = .03; η^2^ = .10), Orienting (F_3,84_ = 4.18; *p <* .01; η^2^ = .13) and Executive Control (F_3,84_ = 5.18; *p <* .01; η^2^ = .16). In children of 3 years old, the attentional effects were lower than in 4-year-olds children (Alerting: F_1,84_ = 7,88; *p <* .01; η^2^ = .09; Orienting: F_1,84_ = 8.16; *p <* .01; η^2^ = .09; Executive control: F_1,84_ = 7.91; *p <* .01; η^2^ = .09) and 5-year-olds children (Alerting: F_1,84_ = 5.63; *p* = .02; η^2^ = .06; Orienting: F_1,84_ = 10.50; *p <* .01; η^2^ = .11, Executive control: F_1,84_ = 13.75; *p <* .01; η^2^ = .14). For Orienting and Executive control effects children of 3 years old had lower ability than 6-year-old children (Orienting: F_1,84_ = 4.18; *p* = .04; η^2^ = .05, Executive control: F_1,84_ = 7.73; *p* < .01; η^2^ = .08). There was no other significant difference (*p* > .05). Figure 4 reports the attentional effects of the four groups of children.

**Fig. 5:**
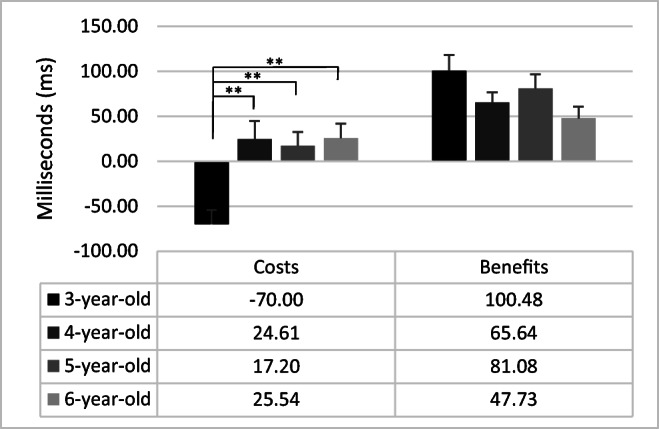
Attentional costs and benefits for each group of children. *Error bars* represent standard errors

The Attentional cost effect (F_3,84_ = 8.04; *p <* .01; η^2^ = .22) revealed that children of 3 years old showed higher costs than children of 4 years old (F_1,84_ = 16.72; *p <* .01; η^2^ = .17), 5 years old (F_1,84_ = 14.21; *p <* .01; η^2^ = .14), and 6 years old (F_1,84_ = 17.05; *p <* .01; η^2^ = .17). There were no other significant differences (F < 1). Attentional benefit effect was not different among group (*p* =.10). Figure 5 reports the attentional costs and benefits in the four groups of children.

### Accuracy analysis

The effects of Group (F_3,84_ = 13.98; *p <* .01; η^2^ = .33), Warning (F_1,84_ = 7.37; *p <* .01; η^2^ = .08), and Executive control (F_1,84_ = 23.72; *p <* .01; η^2^ = .22) were significant. Mean accuracy did not differ between children of 3- and 4-year-old (*p* = .52), while children of 3 years old were less accurate than both children of 5- (F_1,84_ = 29.34; *p <* .01; η^2^ = .26) and 6-year-old (F_1,84_ = 17.44; *p <* .01; η^2^ = .17). Children of 4 years old were less accurate than both children of 5- (F_1,84_ = 22.71; *p <* .01; η^2^ = .21) and 6-year-old (F_1,84_ = 12.43; *p <* .01; η^2^ = .13). Mean accuracy did not differ between children of 5- and 6-year-old (*p* = .22) (See Fig. 2). Participants were more accurate when a warning tone was provided and in congruent than incongruent trials. The main effect of cue type was not significant (F_2,168_ = 1.28; *p* =.28; η^2^ = .01).

The interaction Cue x Group was statistically significant (F_6,168_ = 2.22; *p* = .04; η^2^ = .07).

In all conditions, children of 3 years old were less accurate than children of 5- (invalid: F_1,84_ = 14.04; *p <* .01; η^2^ = .14; no cue: F_1,84_ = 19.42; *p <* .01; η^2^ = .19; valid: F_1,84_ = 32.69; *p <* .01; η^2^ = .28) and 6-year-old (invalid: F_1,84_ = 7.33; *p <* .01; η^2^ = .08; no cue: F_1,84_ = 11.89; *p <* .01; η^2^ = .12; valid: F_1,84_ = 20.40; *p <* .01; η^2^ = .20). Likewise, children of 4 years old were less accurate than children of 5- (invalid: F_1,84_ = 19.17; *p <* .01; η^2^ = .19; no cue: F_1,84_ = 12.42; *p <* .01; η^2^ = .13; valid: F_1,84_ = 19.71; *p <* .01; η^2^ = .19) and 6-year-old (invalid: F_1,84_ = 11.14; *p <* .01; η^2^ = .12; no cue: F_1,84_ = 6.58; *p* = .01; η^2^ = .07; valid: F_1,84_ = 10.49; *p <* .01; η^2^ = .11). Moreover, children of 3 years old were more accurate in invalid trials than both no cue (F_1,84_ = 4.38; *p* = .04; η^2^ = .05) trials and valid trials (F_1,84_ = 12.11; *p <* .01; η^2^ = .13). The was no other significant difference.

### Attentional effects

The ANOVAs on the attentional effects revealed a significant effect of Group for Orienting (F_3,84_ = 3.69; *p* = .01; η^2^ = .12). In children of 3 years old, orienting ability was lower than in children of 4- (F_1,84_ = 4.70; *p* = .03; η^2^ = .05), 5 years (F_1,84_ = 9.22; *p <* .01; η^2^ = .10) and 6-year-old (F_1,84_ = 7.06; *p <* .01; η^2^ = .08). There were no other significant differences (*p* > .05). The difference between groups in Alerting, Executive control, Attentional costs, and benefits were not statistically significant (*p* > .05).

### Target color analysis (reaction time)

The analysis of variance showed a significant main effect of Target Color (F_1,83_ =21.39; *p <*.01; η^2^ =.20). Children were faster when the target was orange than when it was yellow. The following interactions Target Color x Executive Control (F_1,83_ =13.04; *p <*.01; η^2^ =.14) and Group x Target Color x Cue x Executive Control (F_6,166_ =2.50; *p* =.02; η^2^ =.08) were statistically significant. The Group x Target Color x Warning (F_3,83_ =.81; *p* =.49; η^2^ =.03), Group x Target Color x Cue (F_3,83_ =.37; *p* =.89; η^2^ =.01), Group x Target Color x Executive Control (F_3,83_ =.90; *p* =.44; η^2^ =.03) interactions were not statistically significant.

To analyze the significant interactions, a Target Color x Cue x Executive control ANOVA was separately performed for each children’s group. For 3-, 5-, and 6-year-old children, the interactions were not statistically significant. Conversely, 4-years-old children were faster when an orange target than a yellow target was presented in the valid trials of the congruent condition (F_1,21_ =7.88; *p* =.01; η^2^ =.28) and in the no-cue trials of the incongruent condition (F_1,21_ =25.31; *p <*.01; η^2^ =.56).

### Target color analysis (accuracy)

The main effect of Target color (F_1,84_ =.82; *p* =.37; η^2^<.01) was not statistically significant.

The Group x Target Color x Cue x Executive Control (F_6,168_ =2.56; *p* =.02; η^2^ =.08) and Group x Warning x Target Color x Cue x Executive Control (F_6,168_ =2.26; *p* =.04; η^2^ =.07) interactions were statistically significant. To analyze these interactions, a Target Color x Warning x Cue x Executive control was separately performed for each children’s group. For 4-, 5-, and 6-year-old children, the interactions involving Target Color were not statistically significant. 3-year-old children were more accurate when an orange target than a yellow target was presented in the valid trials of the congruent condition (F_1,21_ =7.88; *p* =.01; η^2^ =.27). For 4-year-old, the Target Color x Warning x Cue x Executive control interaction was significant (F_2,42_ =4.51; *p* =.02; η^2^ =.18), but no differences were found for target color in all conditions.

The Group x Target Color x Warning (F_3,84_ =.96; *p* =.41; η^2^ =.03), Group x Target Color x Cue (F_6,168_ =.95; *p* =.46; η^2^ =.03), and Group x Target Color x Executive Control (F_3,84_ =1.87; *p* =.14; η^2^ =.06) interactions were not statistically significant.

### Reliability analysis of the attentional measures from the ANTI-Birds

Table 3 reports Pearson’s correlations of the split-half reliability analysis. Considering RT, the overall score of RT showed acceptable reliability (Spearman-Brown *r* = .98), and the (Executive control effect showed borderline reliability (Spearman-Brown *r* =.76) while the other attentional network scores had problematic reliability (Alerting effect: Spearman-Brown *r* = .04; Orienting effect: Spearman-Brown *r* =.38). These results are in line with previous studies that adopted ANT tasks to assess the attentional networks (Luna et al., 2021; MacLeod et al., 2010; Roca et al., 2018).

**Table 3:** Results of the reliability analyses

	Mean *r*	Spearman test-retest prophecy
Attentional scores (RTs)		
Alerting effect	.02	.04
Orienting effect	.24	.38
Executive Control effect	.62	.76
Overall RT	.96	.98

## Discussion

Our aim in developing a child variant of the ANTI test with non-directional stimuli was to provide an appropriate tool for measuring attentional components in early childhood. Moreover, the test needed to show sensitivity to the development of attention in children aged 3 through 6 years. The results of our study suggest that these aims were met. First of all, all children were able to complete the task, and a significant effect for each attention network was observed. These results support the potential of the task as a tool for experimental and clinical evaluation.

Moreover, according to Posner and Petersen’s model (Petersen & Posner, 2012; Posner & Petersen, 1990), research data confirm different developmental trajectories of attentional networks through the lifespan (e.g., Lewis et al., 2018).

Generally, the study confirms an early acquisition of the skills necessary to complete a complex experimental task (Rueda et al., 2004). Specifically, the development pattern showed that children of 3 years were significantly faster but lower accurate in the responses confirming a trade-off effect, reflecting lower inhibition ability in this age group (Carlson & Wang, 2007). Accordingly, Jones et al. (2003) evidenced that inhibition skills improved by 22% to 90% between 3 and 4 years.

Moreover, children of 3 years of age showed both lower orienting and alerting effects than older children. In particular, smaller alerting scores indicated that young children of 3 years of age benefited less than older children from having an auditory warning signal before the presentation of the target. This is consistent with the view according to which alerting continues to develop during the preschool and early school years (Mezzacappa, 2004; Rueda et al., 2004). For example, using the child ANT, Mezzacappa (2004) showed a trend to larger alerting scores with age in a sample of 5- to 7-year-old children. Developmental changes in alertness during childhood have been generally related to the continuous maturation of frontal systems during this period (Jonkman, 2006; Jonkman et al., 2003). Furthermore, Abundis-Gutiérrez et al. (2014) evidenced that 4-6-years-old children did not show any differences in amplitude of the P1 and P2 peak between tone and no-tone conditions until about 300 ms after the presentation of the tone. This result seems to indicate poor early processing of warning cues in early and middle childhood. Starting at 4 years of age, the presence of a warning tone seems to impact performance positively (i.e., faster reaction times in tone conditions than in no-tone conditions).

On the other hand, children of 3 years also showed smaller orienting scores than the rest of the age groups, suggesting a developmental improvement in orienting and reallocation of attention between 3 and 4 years of age. The analysis of attentional cueing costs and benefits revealed that the reorienting costs of having an invalid spatial cue (invalid minus no-cue conditions) were reduced in children of 3 years of age. In contrast, the benefits of presenting a valid spatial cue (no-cue minus valid conditions) tended to be stable through the age groups. This result is consistent with the view according to which children under 7 years of age are not yet completely efficient when using valid orienting cues to facilitate the processing of the target (Abundis-Gutiérrez et al., 2014). Modifying the ANT to include invalid orienting cues and calculating the score by subtracting valid-cue trials from invalid cue trials provides a measure of orienting that mainly grasps processes related to disengagement and reallocation of attention. According to our data, previous studies indicated that the ability to shift attention to exogenous cues differs little between children and adults while disengaging and reorienting attention seem to improve with age (Brodeur & Enns, 1997; Pozuelos et al., 2014; Trick & Enns, 1998).

Finally, children of 3 years of age showed a smaller conflict effect than older children. This was probably due to their faster responses than the other groups, especially in the incongruent condition. This result may indicate children’s greater difficulties in processing contextual information and modulating behavior during a dominant response. Accordingly, Jones et al. (2003) observed that inhibition skills improve considerably between 3 and 4 years.

Regarding whether the interactions between the attention networks observed in adults are present in preschool children and whether they change with age during this period, we did not find a larger conflict effect under higher alertness conditions. This result has been previously reported in adults and children and interpreted as a negative consequence of high alerting states on performance in detection and conflict resolution tasks (Aston-Jones et al., 1999; Posner, 1978) due to the use of automatic responses instead of more controlled forms of action (Posner, 1994). Conversely, Pozuelos et al. (2014) showed that young children (6 years) had larger interference effects in no-tone conditions when accuracy is considered and explained this result as difficulty in sustained attention in the absence of a warning cue.

Previous studies (Abundis-Gutiérrez et al., 2014; Pozuelos et al., 2014) found a significant Orienting x Executive Control interaction. This result suggested that the appearance of an invalid cue before the target leads to poorer efficiency in resolving a conflictual condition. In our study, this interaction was not significant, and this result indicates that the processing of incongruent information is difficult for children younger than six years, regardless of the facilitation offered by valid orienting cues. This result is in line with Abundis-Gutiérrez et al. (2014) that failed to find a significant interaction in the youngest children (4-6-years-old). Moreover, the fact that no second-order interaction with age was observed suggests that the alerting influence on the executive control is an essential characteristic of the attention system or that the influence of one network over the others develops earlier than the age range tested in our study.

## Limitations

Future studies will benefit from addressing some limitations of the present study. First, a longitudinal design in which one group of children complete the ANTI-birds at several time points is required. Indeed, the present study was cross-sectional and did not control intra-individual differences across time. Second, in the present study, we used a child variant of the original version of the ANTI, originally elaborated by Callejas, Lupianez, and Tudela (2004). However, the flanker task used in our child version of the ANTI (which includes large, brightly colored cartoon birds as flanker stimuli) may not be as effective at producing interference as other, more frequently used flanker tasks (e.g., those with arrows or letters). Therefore, it will be interesting to evaluate the effect of different stimuli on attentional networks in preschool children in a future study. Moreover, the orange target decreased participants’ reaction times, but it affected only the younger groups (3- and 4-years-old). Future studies that will include this task should control the possible effect of the target color. Third, we did not include older children, adolescents, and adults. This has to be kept in mind because our results cannot give information about any additional improvements in the three attentional networks beyond 6 years of age. Lastly, future studies should include a larger sample size to increase the statistical power of the analysis.

## Conclusions

The present experiment is the first to examine the development and the interactions among attention networks in children aged 3 through 6 years of age using the ANTI.

The child variant of the task presented in this study provides a measurement of each attentional network and highlights how attentional functions emerge during the preschool years. In particular, significant changes for alerting, orienting, and executive control networks were observed between 3 and 4 years of age, while no differences were observed between 4 and 6 years. These findings suggest that all attentional abilities greatly improve between 3 and 4 years of age. The results showed that this task is at least as reliable as different versions of the ANT (Luna et al., 2021; MacLeod et al., 2010; Roca et al., 2018) to assess the classic attentional effects. The Alerting and Orienting effect showed problematic reliability, but these results are common when using difference scores (for a discussion, see Hedge et al., 2018).
